# Light-enhanced catalytic activity of stable and large gold nanoparticles in homocoupling reactions

**DOI:** 10.1038/s41598-024-51695-3

**Published:** 2024-01-16

**Authors:** Jian Hou, Jemima A. Lartey, Chang Yeon Lee, Jun-Hyun Kim

**Affiliations:** 1 School of Intelligent Manufacturing, Luoyang Institute of Science and Technology, Luoyang, 471023 China; 2 Department of Chemistry, Illinois State University, Normal, IL 61790-4160 USA; 3 Department of Energy and Chemical Engineering/Innovation Center for Chemical Engineering, Incheon National University, Incheon, 22012 Republic of Korea

**Keywords:** Chemistry, Materials science, Nanoscience and technology

## Abstract

Validating the direct photocatalytic activity of colloidal plasmonic nanoparticles is challenging due to their limited stability and needed support materials that can often contribute to the chemical reactions. Stable gold nanoparticles (AuNPs) with tunable sizes are prepared across porous polymer particles without any chemical bonds where the resulting composite particles exhibit intense surface plasmon resonances (SPRs) in the visible region. These composite particles are then tested as photocatalysts under a broadband solar-simulated light source to examine the contribution degree of photothermal heating and SPR coming from the incorporated AuNPs in the C–C bond forming homocoupling reaction. Generally, the thermal and photothermal heating are the main driving force to increase the reactivity of relatively smaller AuNPs (~ 44 nm in diameter) with a narrower SPR band. However, the SPR-induced catalytic activity is much greater for the composite particles containing larger AuNPs (~ 87 nm in diameter) with a broader SPR. As the polymer particle matrix does not influence the catalytic activity (e.g., inducing charge delocalization and/or separation), the unique SPR role of the colloidal AuNPs in the catalytic reaction is assessable under light irradiation. This study experimentally demonstrates the possibility of evaluating the direct contribution of SPRs to photocatalytic chemical reactions.

## Introduction

Understanding plasmonic metal nanoparticles (NPs) as photo-enhanced catalysts is an ongoing challenge due to many potential variables, including the type of metal toward chemical reactivity, stability, light source, and external support materials (e.g., capping agents or support substances)^1–5^. Particularly, colloidal catalytic systems often require stable plasmonic metal NPs that are well dispersed in solution without causing aggregations under light irradiation to validate their overall catalytic functions. However, many colloidal plasmonic metal NPs exhibit considerably poor stability in most chemical reaction conditions, which makes very difficult to access the degree of catalytic activity primarily induced by light-induced heating event or direct plasmon band excitation process. In this sense, stable support materials and/or capping agents have been introduced to overcome the instability of plasmonic metal NPs (i.e., preventing their aggregation into catalytically inactive bulk metal)^2,6–9^. Although securing colloidal stability can generally improve the catalytic activity of metal NPs, there are a few more important aspects to be considered in photocatalytic applications. For instance, metal NPs are physically integrated onto a support substance (i.e., strong interactions), where the photocatalytic activity of integrated metal NPs can be easily influenced by the light absorption, photothermal heating, and charge separation/migration degree of the substrate^8,10–12^. The synergistical contributions of the substrate to the catalytic activity are highly dependent on the type and structure of support materials, which make somewhat complicated to generalize and examine the photocatalytic properties of the colloidal plasmonic metal NPs. In addition, these support materials often limit the structural control of the integrated metal NPs. On the other hand, the utilization of capping agents often allows for controlling the final structures (i.e., size and shape) of the metal NPs^13–17^. However, the presence of the capping agents around metal surfaces could promote the catalytic selectivity but generally decrease the reactivity (i.e., blocking catalytically active sites).

Here, we employ a porous polymer particle as a support to physically incorporate metal NPs without any electrostatic or chemical interactions. As the polymer particles with loosely crosslinked networks do not have strong attractive forces with the metal NPs, the systematic growth of the integrated metal NPs can be achieved. These stable plasmonic metal NPs exhibit intense and broad surface plasmon resonance (SPR) patterns due to their distinctively controlled size distributions, where the direct photocatalytic activity can be evaluated as a function of the SPR bands without considering the effect of the polymer particle substrate. Specifically, it is noted that the polymer particles can be maintained in a fully swollen structure so as not to influence light absorption capability, photothermal heating, or charge separation/migration to the integrated metal NPs under light irradiation. Excluding these contributions, the composite particles allow for precise evaluation of the direct photocatalytic property of integrated plasmonic metal NPs possessing unique SPR bands in a chemical reaction process.

Specifically, plasmonic gold nanoparticles (AuNPs) were reliably prepared and grown across the poly(*N*-isopropylacrylamide), PNIPAM, particles via a seed-growth method^18–20^. The loosely crosslinked PNIPAM particles readily allowed for the systematical growth of the integrated AuNPs up to sub-100 nm in diameter to possess strong SPR bands. The series of resulting composite particles was tested as photocatalysts under a solar-simulated light source. Among many catalytic reactions, including oxidation, reduction, and dye degradation, a challenging C–C bond forming reaction was chosen in this study. Unlike other chemical reactions, this type of reaction requires relatively harsh conditions and a long reaction time. Thus, overall stability and photocatalytic activity can be precisely validated as a function of the size and SPR band of the integrated AuNPs under light irradiation. In addition, the main photocatalytic enhancement by a broadband light source that can cover both the interband or SPR regions of plasmonic metal NPs could offer much greater practicability to design chemical reaction systems^21,22^, instead of utilizing one specific or narrow wavelength of light. Thus, the use of a sunlight source in this study can not only demonstrate how the plasmonic AuNPs exhibit photocatalytic properties in the carbon bond forming reaction but also be highly simple to establish reaction conditions to improve the overall reaction yields. Understanding the photocatalytic functions of the composite particles possessing structurally regulated plasmonic metal NPs will eventually lead to the development of industrially practical chemical transformation reaction systems.

## Experimental

### Materials

Gold salt (HAuCl_4_·3H_2_O), trisodium citrate dihydrate, *N,N’*-methylene-bis-acrylamide (BIS), ammonium persulfate (APS), potassium carbonate (K_2_CO_3_), phenylboronic acid, cetyltrimethylammonium bromide solution (CTAB), L-ascorbic acid (ASA), and ethanol (EtOH) were purchased from Fisher Scientific. N-isopropylacrylamide (NIPAM) was purchased from Sigma-Aldrich and recrystallized in hexanes prior to use. Water used in all reactions was obtained from a Nanopure water system equipped with a 0.2 µm membrane filter (Barnstead/Thermolyne). All glassware was cleaned with a strong acid (3:1 ratio of HCl:HNO_3_) and a base (KOH in isopropanol).

### Preparation of AuNP seed-PNIPAM particles

Poly(N-isoprpoylacrylamide), PNIPAM, particles were prepared by conventional free radical polymerization using NIPAM (1.00 g), BIS (0.10 g), and APS (0.12 g) in 200 mL water. After the polymerization at 70 °C under argon gas for 5 h, the polymer solution was filtered through a Whatman grade 1 filter paper to remove polymer aggregates. Subsequently, 10 mL of the PNIPAM solution was mixed with an aliquot of 1 wt% gold salt solution (0.2 mL) in a 20 mL glass vial, which was placed in a water-jacketed beaker. The mixture was stirred for at least 30 min to completely disperse gold ions across the PNIPAM particles. After adding an aliquot of 1 wt% trisodium citrate (1.0 mL) to the mixture, a solar-simulated light equipped with a continuous Xe arc lamp and air mass filter (provides ~ 100 mW/cm^2^, Newport Corporation) was illuminated for 3 h. This light-enhanced synthetic method effectively allowed for the formation of small gold nanoparticles (AuNPs) across the PNIPAM particles. The resulting AuNP-integrated PNIPAM particles were isolated by multiple centrifugation steps (6000 rpm × 1, 5000 rpm × 1, 4000 rpm × 1, 3000 rpm × 1, and 2000 rpm × 1 for 30 min) to remove free AuNPs. The final precipitate was resuspended in 10 mL water prior to use as AuNP seed-PNIPAM particles.

### Preparation of composite particles containing grown AuNPs

An aliquot (0.6 mL) of 1 wt% gold salt solution was added to 30 mL of a preheated CTAB solution (100 mM, 0.036 g/mL) over 32 °C in a 50 mL centrifuge tube. L-ascorbic acid (0.6 mL of 100 mM) was then introduced to the mixture, which changed the color of the solution from homogeneous orange to clear. After adding various amounts of the AuNP seed-PNIPAM particles (0.025–1.75 mL) to the several clear solutions, the centrifuge tubes were placed under a solar-simulated light for 40 min. The color of the solution developed from pinkish red to bright purple and brown. The final mixture was centrifuged at 2000 rpm for 15 min twice to remove unreacted species, and the precipitate was suspended either in water or EtOH to possess the extinction of 6. This process reliably grew the integrated AuNP seeds (i.e., sub-10 nm) to relatively larger sizes across the PNIPAM particles to possess intense and broad SPR bands.

### Homocoupling reaction using various composite particles in the presence and absence of a broadband light source

The dispersed composite particles in EtOH (3.0 mL) were added to a glass vial containing phenylboronic acid (21 mg, 0.17 mmol) and K_2_CO_3_ (67.0 mg, 0.48 mmol). The vial was immersed in a water-jacketed beaker or preheated oil bath (e.g., 40 °C), where the reaction mixture was continuously stirred in the presence and absence of a solar-simulated light source (~ 100 mW/cm^2^). After the reaction, an aliquot of the reaction mixture (0.6 mL) was transferred to an Eppendorf tube for microcentrifugation (10,000 rpm × 5 min). The top EtOH layer (0.5 mL) was then subjected to GC analysis. To examine the recyclability and stability, the composite nanoparticles after the reaction in an oil bath were recovered by several centrifugation and purification steps using a mixture of water and EtOH. The purified composite particles were then resuspended EtOH and recycled under the same homocoupling reaction.

### Characterization

A scanning electron microscope (SEM, FEI-Quanta 650) and a transmission electron microscope (TEM, Hitachi H8100) were used to examine the size distribution of PNIPAM particles and the incorporated AuNPs. Scanning transmission electron microscope (STEM, HAADF − STEM, FEI Tecnai G2 F30 SuperTwin operating at 300 kV) and energy dispersive X-ray (EDX) were also employed to obtain the high-angle annular dark-field images and elemental maps of the representative composite particles. The particle samples for the SEM and TEM analyses were deposited and dried on a silicon wafer and 300 mesh carbon-coated copper grids, respectively. Dynamic light scattering (DLS, ZetaPALS, Brookhaven Instruments Corp.,) equipped with a 35 mW laser was used to examine the hydrodynamic diameter of the particles after diluting them (× 20 times) in either water or EtOH. The surface charges were also collected after suspending the composite particles in water and EtOH (~ 0.05 mg/mL). All data were an average of at least five measurements. An Agilent UV–Visible spectrometer equipped with a temperature controller was used to characterize the optical properties (i.e., surface plasmon resonance-SPR) of the composite nanoparticles. All particle samples were suspended in water or EtOH in a quartz cell for the measurement. The reversible changes of SPR bands above and below the lower critical solution temperature (LCST) were performed by controlling the solution temperature from 20 °C to 45 °C). A thermogravimetric analyzer (TGA, SDT Q600, TA Instrument) was used to monitor the weight loss patterns of bare PNIPAM and composite particles as a function of temperature. All particle sample were completely dried by a rotary evaporator and an oven overnight. A small quantity of the dried particles (3–5 mg) was placed in an alumina crucible, and their thermal decomposition was monitored under an N_2_ gas environment. An atomic absorption spectrophotometer (AAnalyst 200, Perkin Elmer) was also used to determine the amount of integrated AuNPs across the PNIPAM particles. A strong acid (1.0 mL of a 1:1 v/v ratio of HCl to HNO_3_) was added to the composite particle solution (0.1 mL) to dissolve the integrated AuNPs for 10 min. The final mixture was briefly sonicated after adding water (8.9 mL) and centrifuged (4000 rpm × 30 min) to collect the aqueous layer for the analysis. The total amount of Au atoms in the composite particles was compared to the gold ICP standard solutions (Sigma-Aldrich) via the Beer − Lambert law. The final value was based on the average of three minimum measurements using three different batches. Similarly, an inductively coupled plasma atomic emission spectrometer (ICP-AES, Optima 8300, PerkinElmer) was employed to examine the possible leaching of gold species from the polymer particles during the catalytic reaction. After the catalytic reaction, top EtOH solution (1.0 mL) was collected, completely dried, and resuspended in the strong acid solution (1.0 mL). The resulting solution was diluted with water (9.0 mL) for ICP analysis (average of three measurements). Among three emission lines, the 242.795 nm wavelength with the highest calibration sensitivity and accuracy was compared to the standard curve to analyze the leaching amount of gold species. A powder X-ray diffraction (PXRD, MiniFlex 600, Rigaku Corp.) was employed to examine the crystallinity and size distribution of integrated AuNPs across PNIPAM particles under the following conditions (CuKα x-ray, scan range: 5–80°, 0.02 steps, and 2°/min). An aliquot of concentrated composite particle solution was deposited and dried on a glass slide several times. Gas chromatography (Focus GC, Thermo Scientific) equipped with a fused silica capillary column (30 m) and a flame ionization detector was used to examine the photocatalytic homocoupling yields.

## Results

**Figure 1:**
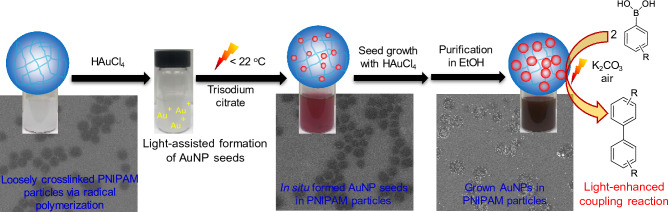
The overall preparation process of composite particles consisting of AuNPs across loosely crosslinked PNIPAM particle network.

Colloidal metal nanoparticles (NPs) that respond to light can offer photocatalytic potential for various chemical reactions. Here, plasmonic gold nanoparticles (AuNPs) were prepared and grown across porous PNIPAM host particles via a reduction and growth method (Fig. 1). This in situ process enabled us to systematically prepare various sizes of integrated AuNPs in a controlled manner. Unlike other host substrates, the hydrophilic PNIPAM particles with a loosely crosslinked network readily overcome the limitations of size and structure turnability of the guest NPs^6,23–25^. Thus, the incorporated AuNPs with controlled sizes in the polymer particles exhibit strong surface plasmon resonance (SPR) bands in the visible area. As the resulting composite particles possess relatively large sizes of AuNPs, SPR-dependent direct photocatalytic activity can be examined under a broadband light source (i.e., solar-simulated light). Given the absence of interfacial interactions and/or possible assistance of charge separation process, the PNIPAM-derived host substrates do not influence the overall catalytic property of the integrated AuNPs. This unique feature allowed for the validation of the main photocatalytic activity of the colloidal plasmonic AuNPs. Specifically, the degree of catalytic property changes under light irradiation without considering instability concerns and substrate contributions to chemical reactions could be directly correlated to the SPR patterns of the integrated plasmonic AuNPs.

**Figure 2:**
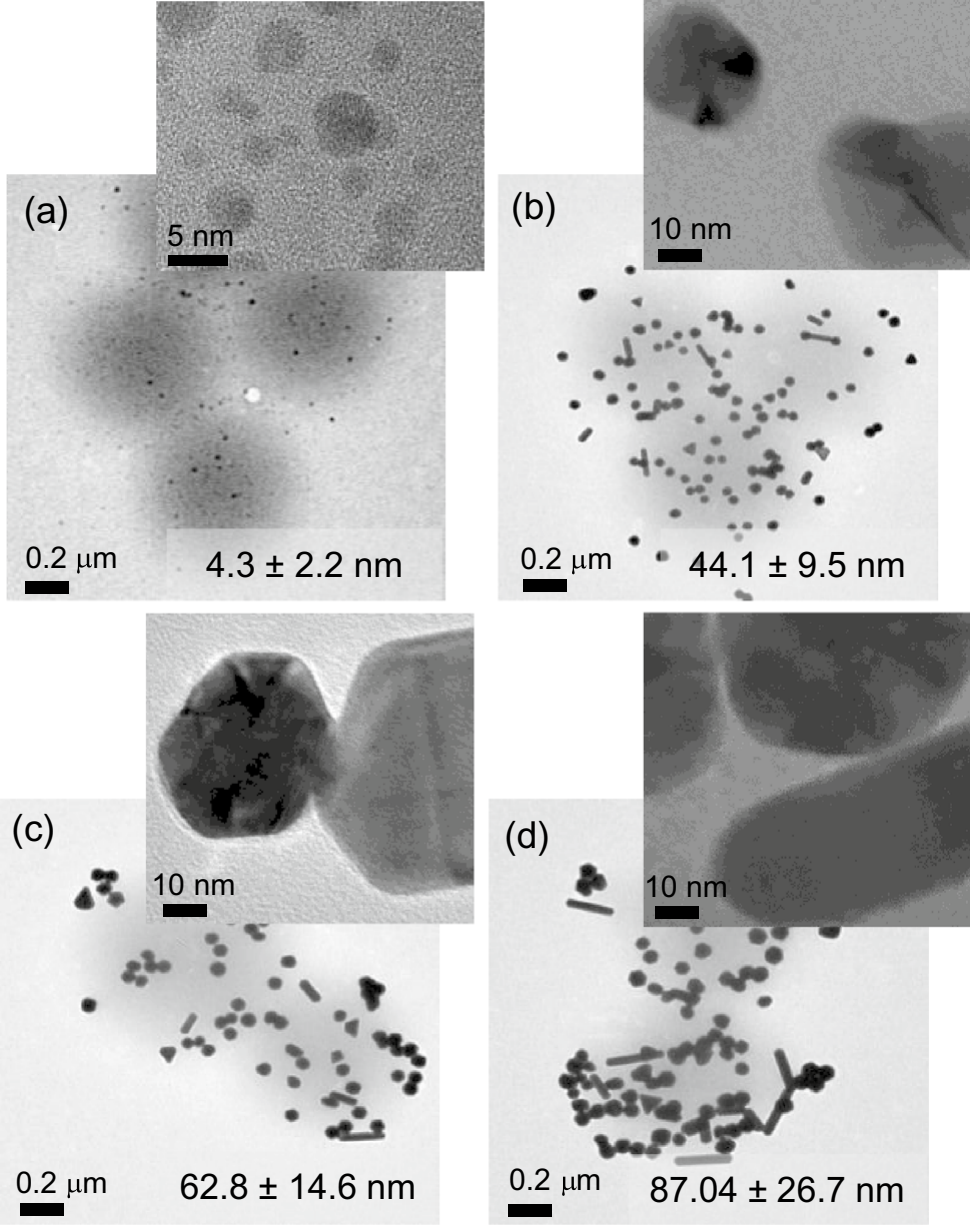
TEM images of (a) AuNP seeds and grown AuNPs in PNIPAM particles using a different amount of (**b**) 0.75 mL, (**c**) 0.25 mL, and (**d**) 0.075 mL AuNP seed solution (the average diameter of AuNPs was estimated by the ImageJ software).

Initially, small AuNPs were effectively formed across the swollen PNIPAM particles in situ under our light-induced environments (Fig. 2 and Supplementary Fig. S1). The integrated sub-10 nm AuNPs (Fig. 2a) were used as seeds to steadily grow much larger AuNPs (Fig. 2b–d). Although it has been reported that the light-induced reduction method could promote aggregation of even small AuNPs^26^, the use of PNIPAM-based host polymer particles readily avoids this problematic process. Thus, simply adjusting the amounts of the seed and the growth solution precisely controlled the final size of the incorporated AuNPs (from ~ 44 nm to ~ 87 nm in diameter shown in the TEM images) without aggregations. Generally, the size and distribution of the integrated AuNPs were notably increased by decreasing the amount of the AuNP seed solution. As the size of the AuNPs increased, lattice fringes gradually disappeared to display different crystalline domains (i.e., polycrystalline feature) containing a spectrum of defects (e.g., stacking, twinning, multiple grain boundaries)^27–29^. A further decrease of the seed solution below 75 µL resulted in the outgrowth of the integrated AuNPs causing the severe aggregations/precipitation observed by the SEM images (Supplementary Fig. S2). This detectable instability of the composite particles was also confirmed by the disappearance of the SPR peaks. On the other hand, increasing the amount of the composite seed solution over 750 µL somewhat limited the growth of the integrated AuNPs, whose SPR patterns remained almost unchanged, which will be discussed in more detail below. It is noted that the size of the integrated AuNPs prepared in this study is distinctively large enough to maximize the harvesting efficiency of broadband light (i.e., strong SPRs) for use as photocatalysts. To confirm the successful in situ growth of the integrated AuNP seeds, elemental maps of the representative composite particles (i.e., grown AuNPs in PNIPAM particles using 0.75 mL of the seed solution) were obtained using STEM-EDX (Supplementary Fig. S3). The distribution of AuNPs was mostly across the PNIPAM particles without noticeable free AuNPs.

**Figure 3:**
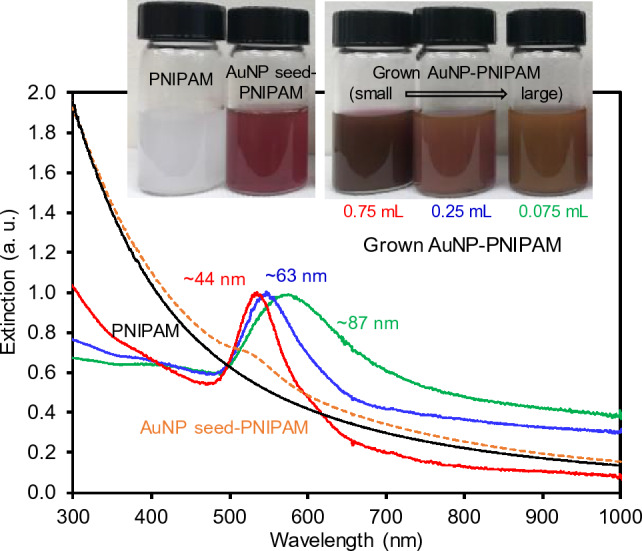
Digital photos and normalized SPR patterns of PNIPAM, AuNP seed-PNIPAM, and grown AuNPs in PNIPAM particles.

The extinction spectra of the PNIPAM, AuNP seed-PNIPAM, and systematically grown AuNP-PNIPAM particles were thoroughly examined (Fig. 3). The bare PNIPAM exhibited a typical scattering pattern from the UV to visible ranges without any distinctive absorbance^30,31^. The AuNP-seed PNIPAM particles displayed an almost identical absorption pattern except for the appearance of a detectable SPR peak at 520 nm, strongly suggesting the presence of small plasmonic AuNPs. The composite particles with the grown AuNPs possessed significantly intense plasmonic bands, whose overall positions and widths were notably influenced by the size and distribution of the integrated AuNPs. Specifically, the grown AuNPs possessed the following SPR peak positions: ~ 44 nm AuNPs at 536 nm, ~ 63 nm AuNPs at 547 nm, and ~ 87 nm AuNPs at 573 nm, while their band widths gradually increased, presumably due to the polydispersity. To confirm the integration of AuNPs into the PNIPAM particles, the reversible SPR bands of the composite particles were examined above and below the lower critical solution temperature (LCST) (Supplementary Fig. S4). The deswelling of the polymer particles above the LCST effectively induced a closer proximity for the integrated AuNPs, resulting in the plasmonic coupling. Upon cooling below the LCST, the composite particles regained their original swollen state, leading to the recovery of the SPR bands. These reversible SPR patterns evidently indicated the successful integration of large AuNPs across the PNIPAM particle networks. These intense and broad SPR patterns of these integrated AuNPs are highly suitable for the investigation of photocatalytic activity in a chemical reaction under light irradiation.

Separately, the amount of integrated AuNPs was estimated by atomic absorption spectroscopy (AAS) and thermogravimetric analyzer (TGA). The AA analysis of gold atoms after dissolving the integrated AuNPs with strong acid resulted in nearly 0.14 mg/mL for the seed and 1.26–3.22 mg/mL for the grown AuNPs. The TGA analysis clearly revealed that the weight loss was much less for the composite particles containing larger AuNPs (Supplementary Fig. S5). While the PNIPAM particles started to decompose at ~ 380 °C, the integrated AuNPs appeared to be thermally stable and mostly remained over 480 °C. Significantly higher residues were attributed to the amount of the integrated AuNPs with respect to the mass of the most PNIPAM particles. These weight loss patterns evidently indicated the successful preparation of composite particles containing different amounts of AuNPs across the PNIPAM particles.

**Figure 4:**
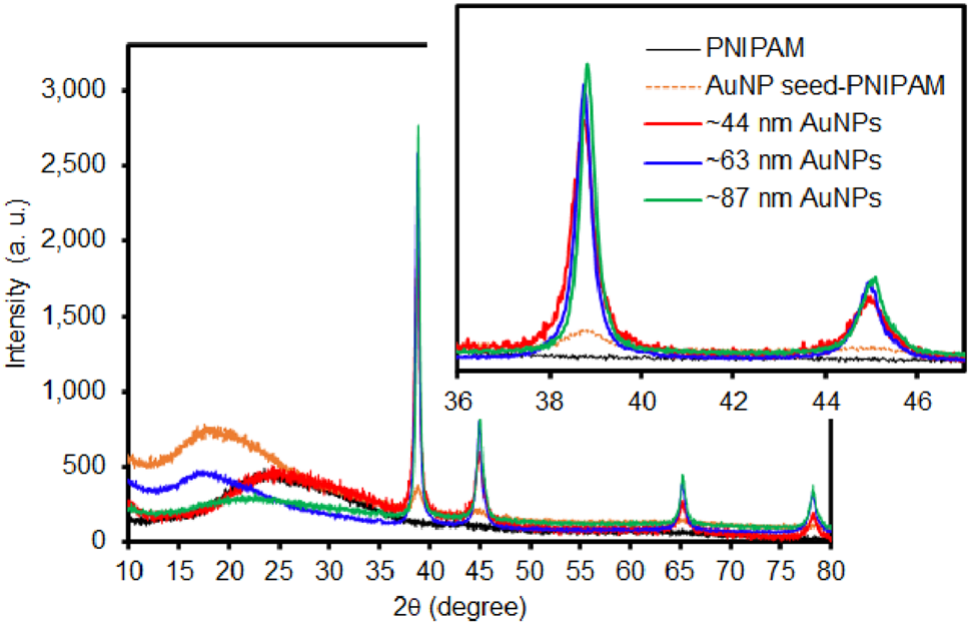
PXRD patterns of PNIPAM, AuNP seed-PNIPAM, and grown AuNPs across PNIPAM particles.

As the crystallinity of metal NPs greatly influences their catalytic activity^28^, the powder x-ray diffraction (PXRD) patterns of bare PNIPAM and composite particles were collected (Fig. 4). While the bare PNIPAM particles only show a broad peak below 30°, the composite particles displayed four additional peaks at 2θ = 38.3°, 45.2°, 65.5°, and 78.1° for the (111), (200), (220), and (311) planes with space group Fm3m^32–34^, indicating the presence of AuNPs with a face-centered-cubic crystalline structure. The composite particles containing small AuNP seeds showed relatively broad peaks, whereas the composite particles with the grown AuNPs displayed distinctively sharp peaks. Simply examining the peak width and position of the (111) plane can also reveal the size of the AuNPs using the Debye–Scherrer equation (Supplementary Eq. S1)^35,36^, associated with the size-dependent X-ray line broadening pattern of crystalline metal NPs. The example calculations for the size of the integrated AuNPs are also available in the Supplementary Information section. As the size of the incorporated AuNPs increased, it was evident that the band width was inversely proportional (i.e., narrower). Given the high polydispersity of the AuNPs, the calculated size was somewhat different from those TEM images. It has also been reported that the surfaces of larger AuNPs possess presumably more defects due to the inherent deficiencies generated by the synthetic methods^37,38^. Large AuNPs typically exhibit the major facet on the (111) domain, while the other crystallographic planes are presumably coming from different structural defects, including twin/grain boundary, kink, edge dislocation, stepped surface, and island. These discrete crystalline domains could be related to the final size and shape of the AuNPs and play an important role in their overall catalytic activities. As such, the diffraction intensity ratio of the (111)/(200) peaks was obtained to examine the preferential crystallization of the AuNPs. The peak intensity ratio of the composite particles containing the AuNP seeds was ~ 1.81, which was close to the conventional AuNPs (~ 1.90)^33,39,40^. However, all composite particles possessing the grown AuNPs showed much higher peak ratios (3.25–3.40), indicating the preferential growth of the integrated AuNPs in the (111) plane.

**Figure 5:**
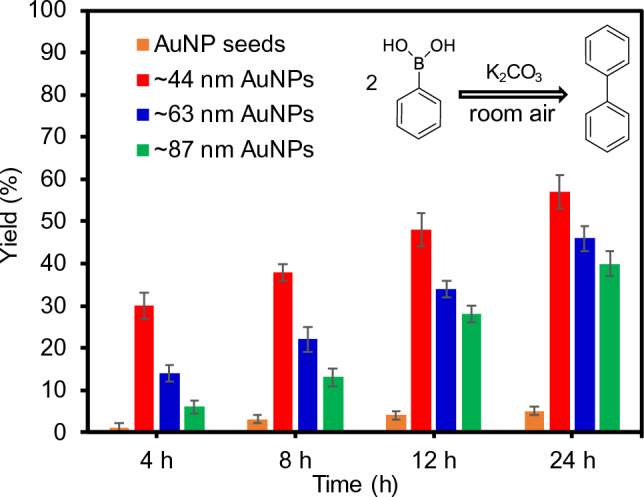
Homocoupling reaction yields using various AuNP-PNIPAM composite particles at ambient conditions as a function of time.

After validating the successful preparation and structural features, the catalytic activity of these composite particles was examined using a C–C bond forming reaction in EtOH as a function of time (Fig. 5). The composite particles possessing the AuNP seeds resulted in notably low yields given their significantly small amounts across the PNIPAM particles. The use of the composite particles containing the grown AuNPs led to much higher reactivity, and the overall conversion yields gradually decreased with increasing the size of the AuNPs. This inverse relationship was expected between the reactivity and size of the grown AuNPs, where the composite particles possessing smaller AuNPs (i.e., ~ 44 nm) resulted in the highest yields. The degree of reaction yield generally slows as a function of time, probably due to the consumption of the reactants. As the instability of the incorporated AuNPs could result in a gradual reduction in the reaction rate over time, the composite particles were recycled two additional times under the same reaction conditions (Supplementary Fig. S6). Overall reaction yields remained almost unchanged even after the third cycle, which suggested the good stability of the incorporated AuNPs (i.e., no notable aggregation) by the host PNIPAM particles to maintain their catalytic activities. This experimental observation suggested that the gradual reduction in the reaction rate over time was mainly influenced by the reactant concentration rather than the instability of the AuNPs.

It is noted that all reactions were carried out at ambient conditions without any external stimulus (e.g., temperature). Although size-dependent catalytic activity and selectivity have been reported for plasmonic NPs, our reaction conditions in pure EtOH solely resulted in the formation of the target compound without any by-products, offering a high degree of simplicity to examine the SPR-driven photocatalytic reactivity^41–43^.

**Figure 6:**
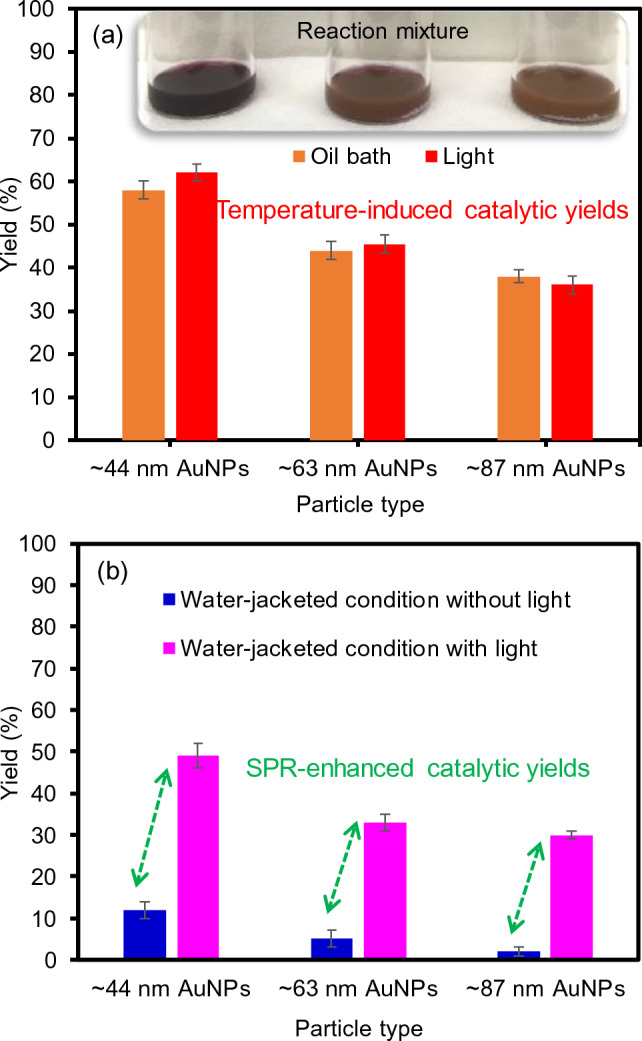
Homocoupling reaction yields in the presence of various AuNP-PNIPAM composite particles using (**a**) an oil bath vs light irradiation and (**b**) a water-jacked beaker at ~ 20 ℃ with and without the solar-simulated light irradiation.

To precisely examine the direct photocatalytic activity of these AuNPs under a broadband light irradiation, the same homocoupling reaction was carried out under one-sun conditions (e.g., ~ 100 mW/cm^2^) using a solar-simulated light source (Fig. 6). The light-induced reaction resulted in the comparable yields to those of the homocoupling reaction at ~ 40 °C in the oil bath, regardless of the size of the grown AuNPs (i.e., temperature-assisted reaction). These reaction yields after 4 h were mainly attributed to the overall temperature of the reaction medium simply due to light-induced heating rather than the direct SPR-induced activity of the AuNPs (Fig. 6a). To monitor the light-induced heating efficiency, the solution temperature of two vials containing pure EtOH and the representative composite particles (i.e., ~ 43 nm AuNPs) was simultaneously collected under the solar-simulated light irradiation as a function of time (Supplementary Fig. S7). As the EtOH possesses a lower heat capacity, the solution temperature quickly reached to ~ 37 °C in 6 min and mostly remained at this saturation temperature. The temperature change of the EtOH medium containing the composite particles was slightly faster (5 min) and higher (~ 41.5 °C). Although the main temperature elevation was possibly associated with the heat capacity of the solvent and glass vial container, the discernably higher temperature profiles of the reaction medium containing the composite particles were evidently contributed by the presence of the plasmonic AuNPs. Although these higher solution temperatures originated from the plasmon-induced heating of the AuNPs under light irradiation, the surface temperature of the AuNPs could be higher than the average solution temperature^44–47^. Furthermore, the final solution temperatures of the composite particles containing ~ 63 nm and ~ 87 nm AuNPs were evaluated to be ~ 43 °C and 46 °C, respectively. These composite particles possessing broader SPR bands exhibited slightly higher photothermal heating capabilities (i.e., beneficial for utilizing sunlight to convert light-to-heat)^47,48^. As such, the increased solution temperatures via light-induced heating could play an important factor in the overall reaction yields of the homocoupling reaction. It is also worth mentioning that the use of the alcohol solvent readily eliminated the temperature-responsive property of the PNIPAM particle matrix (Supplementary Fig. S8), where the fully swollen structure was maintained (i.e., the absence of polymer matrix interferences) during the catalytic process of the incorporated AuNPs regardless of the reaction conditions. Although the incorporated AuNPs could be released from the PNIPAM particles and engaged in the catalytic reaction, the top solution of the mixture after the reaction was subjected to the ICP-AES analysis (Supplementary Fig. S9). The loss of gold species was examined to be nearly trace levels (0.11–0.15 ppm) regardless of the size of integrated AuNPs. This insignificant leaching could marginally affect the overall catalytic property of the composite particles under our reaction conditions.

To minimize the influence of solution temperature on yields, the same reaction was carried out in the water-jacked beaker for 4 h under the light irradiation source (Fig. 6b). This experimental setup readily maintained the constant temperature of the reaction medium (~ 20 °C) with and without the light irradiation source for the entire time. While the yields of the reaction were significantly low without the light irradiation, detectably increased yields were observed with the light irradiation. These improved yields were evidently caused by the SPR-induced photocatalytic property of the composite particles. Specifically, the use of larger AuNPs (~ 87 nm) resulted in the formation of the biphenyl product at trace levels (~ 2%) without the light irradiation but considerably increased the reaction yields (~ 30%) under light irradiation. As the solution temperatures of both reactions were maintained at ~ 20 °C, these distinctively different yields were mainly affected by the light-induced SPR excitation of the AuNPs. Similarly, the use of smaller AuNPs (~ 44 nm and ~ 63 nm) resulted in slightly higher reaction yields (~ 9% and ~ 5%) without the light irradiation, which further increased to ~ 49% and ~ 33% under light irradiation. These experimental results were presumably due to their SPR-induced photocatalytic activities. The SPR excitation process could lead to the generation of dynamic hot electrons (e^−^) and holes (h^+^), where the electrons directly interact with the molecules in close proximity to the AuNP surfaces^42,49,50^. It is also important to remember that the organic-based PNIPAM particles serving as a support do not contribute to the common charge separation process on the surface of the integared AuNPs to improve the catalytic reaction yields (i.e., the elimination of the contribution of support materials). As such, these different reaction yields could be attributed largely to the photocatalytical activity of the plasmonic AuNPs, where the degree of “direct” SPR-enhanced activity was far greater for the relatively larger AuNPs possessing a broader SPR band.

**Scheme 1:**
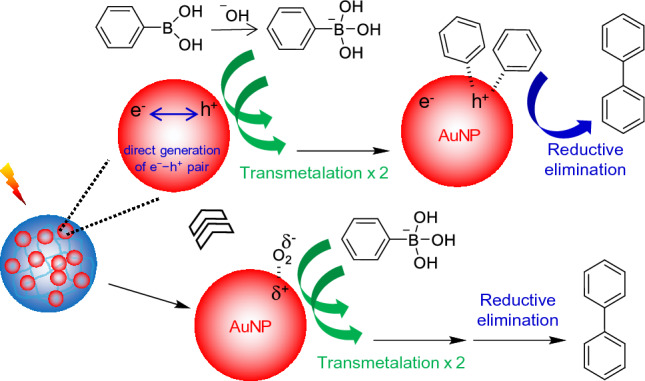
Plausible reaction mechanism under light irradiation.

This SPR-enhanced catalytic process might induce a slightly different reaction pathway for the homocoupling reaction (Scheme 1). The reaction mechanism typically involves the adsorption of molecular oxygen on the surface of the AuNPs where the positively polarized areas attract the hydroxyboronate anion to mainly enhance the transmetalation step^51–54^. In addition to the traditional process, this direct photocatalytic process could generate a number of hot electron (e^−^) and hole (h^+^) pairs across the AuNPs under our reaction conditions, where the hole areas could then contribute to the transmetalation step (i.e., adsorption of hydroxyboronate anions). To prove this speculation, the homocoupling reaction mixture containing the composite particles (i.e., ~ 44 nm AuNPs) was purged with argon gas for 30 min and exposed to the light source. Unlike the typical reaction setup at ambient conditions with abundant molecular oxygen, this experiment eliminated the possible adsorption of oxygen onto the surface of AuNPs, but still resulted in detectable yields (~ 5% in 4 h) under light irradiation. This observation may have suggested that the electron–hole pairs generated under light irradiation could participate in the direct photocatalytic process. However, the traditional reaction pathway associated with the adsorption of molecular oxygens evidently appeared to be still favorable in the homocoupling process. In the absence of the light source, the homocoupling reaction under the argon environment (i.e., absence of molecular oxygen) resulted in the formation of biphenyl at very low levels (< ~ 2%) regardless of the size of AuNPs, even at a mild reaction temperature (e.g., 40 °C). These detectable yields with the grown AuNPs seemed to be still higher than the conventional AuNP-based catalysts prepared by trisodium citrate (e.g., trace levels), possibly due to the residual CTAB around the integrated AuNPs. Although the presence of CTAB could partially polorize the surfaces of the AuNPs to induce the transmetallation of phenylboronic acid^55–57^, we speculated that this contribution was still minimal as the composite particles were washed and dispersed suspended in EtOH (known as a good solvent to remove the surfactant)^58–60^. To support the negligible contribution of CTAB in the catalytic process, the Zeta potentials of the composite particles showed nearly neutral values after the EtOH washing step (Supplementary Fig. S10). More in-depth mechanism studies are underway.

**Figure 7:**
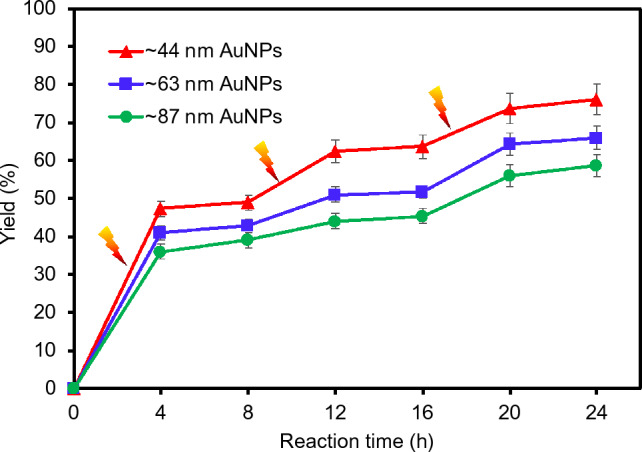
Homocoupling reaction yields using various AuNP-PNIPAM composite particles at ambient conditions with and without the solar-simulated light irradiation as a function of time.

The same homocoupling reaction was carried out on a larger scale to examine the SPR-driven catalytic activity of three composite particles at ambient conditions with (4 h) and without (4 h) the solar-simulated light irradiation (Fig. 7). The yield slopes were generally sharper with the light irradiation, but the yields of reactions marginally increased (e.g., < 4%) in the absence of the light source. Particularly, a much faster rate of reaction was observed under light irradiation after the first 4 h (i.e., over 35% yields), presumably due to the initial concentration of abundant reactant. The rate of reaction gradually decreased (e.g., 5–12% yields) as this type of homocoupling generally follows the first order kinetic^61–63^. This on-and-off trend of the reaction yields clearly indicated the importance of plasmonic AuNPs that can induce both photothermal heating of the reaction medium and SPR-enhanced catalytic activity under light irradiation. It is noted that the reaction conditions tested were highly practical designing other types of chemical reactions, including Ullmann, Suzuki, and Sonogashira couplings without any electrical or thermal input. As such, the composite particles integrated with plasmonic metal NPs can exhibit enhanced photocatalytic properties under a broadband light source, which can potentially serve as alternatives for relatively toxic palladium and expensive platinum-based systems.

## Conclusion

The use of highly porous PNIPAM particles allowed for the reliable preparation of physically integrated AuNPs possessing broad SPR bands. Simply controlling the ratio of the AuNP seed-PNIPAM particles to the growth solution systematically controlled the formation of much larger AuNPs. The resulting stable composites were then used as photocatalysts in the homocoupling of phenylboronic acid under a broadband solar-simulated light, where the solution temperature (e.g., photothermal and electrical thermal input) was the main driving force to result in high yield reactions. Generally, relatively smaller AuNPs (~ 44 nm) integrated into the PNIPAM particles exhibited higher catalytic activity in the presence and absence of heat sources. However, the SPR-driven activity was particularly increased for the composite particles containing relatively larger AuNPs (~ 63 nm and ~ 87 nm) under light irradiation due to the better light harvesting capability. Given the absence of support material contributions to the reaction process, the degree of direct SPR-induced catalytic activity was precisely monitored at a constant reaction temperature (~ 20 °C) under the broadband light source. This empirical test demonstrated the relationships between the SPR bands and the catalytic activity of the composite particles possessing various sizes of AuNPs. Additional studies associated with the reaction pathway and on/off light irradiation clearly suggested the importance of the SPR bands of plasmonic AuNPs in the photocatalytic reaction process. Thus, the ability to control the structural features of plasmonic NPs and understand their catalytic role under light irradiation can lead to the development of highly practical catalytic systems for various chemical transformation reactions.

### Supplementary Information

Supplementary Information.

## Data Availability

All data collected and analyzed in the course of this study are included in this manuscript and its supplements. Any supporting data for this article is available from the corresponding author upon reasonable request.
