# Supplementary material for: Light-enhanced catalytic activity of stable and large gold nanoparticles in homocoupling reactions
Source: Sci Rep. 2024 Jan 16;14:1352. doi: 10.1038/s41598-024-51695-3 (PMC10791751; doi:10.1038/s41598-024-51695-3)
Supplement: Supplementary file 1 — Supplementary Information. [file 41598_2024_51695_MOESM1_ESM.pdf]

Light-Enhanced Catalytic Activity of Stable and Large Gold Nanoparticles in Homocoupling Reactions

Jian Hou[1†], Jemima A. Lartey[2†], Chang Yeon Lee[3,*], Jun-Hyun Kim[2,*]

[1]School of Intelligent Manufacturing, Luoyang Institute of Science and Technology, Luoyang 471023, China

[2]Department of Chemistry, Illinois State University, Normal, IL 61790-4160, USA

[3]Department of Energy and Chemical Engineering, Incheon National University, Incheon 22012, Republic of Korea

[†]These authors contributed equally to this work.

*cylee@inu.ac.kr (ORCID #: 0000-0002-1131-9071) and *jkim5@ilstu.edu (ORCID #: 0000-0001-8145-6269)

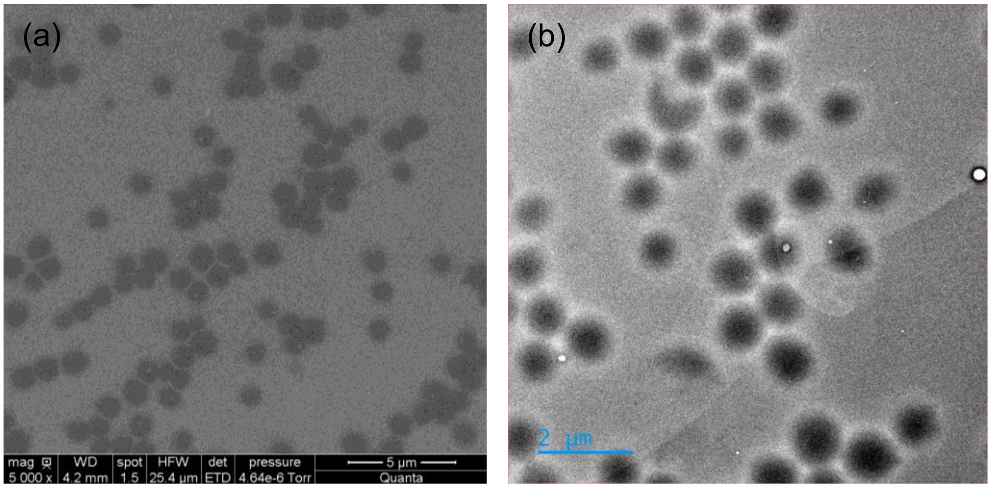

Figure S1. SEM (a) and TEM (b) images of bare PNIPAM particles (note: slightly under-focused TEM image due to the poor contrast of the polymer particles).

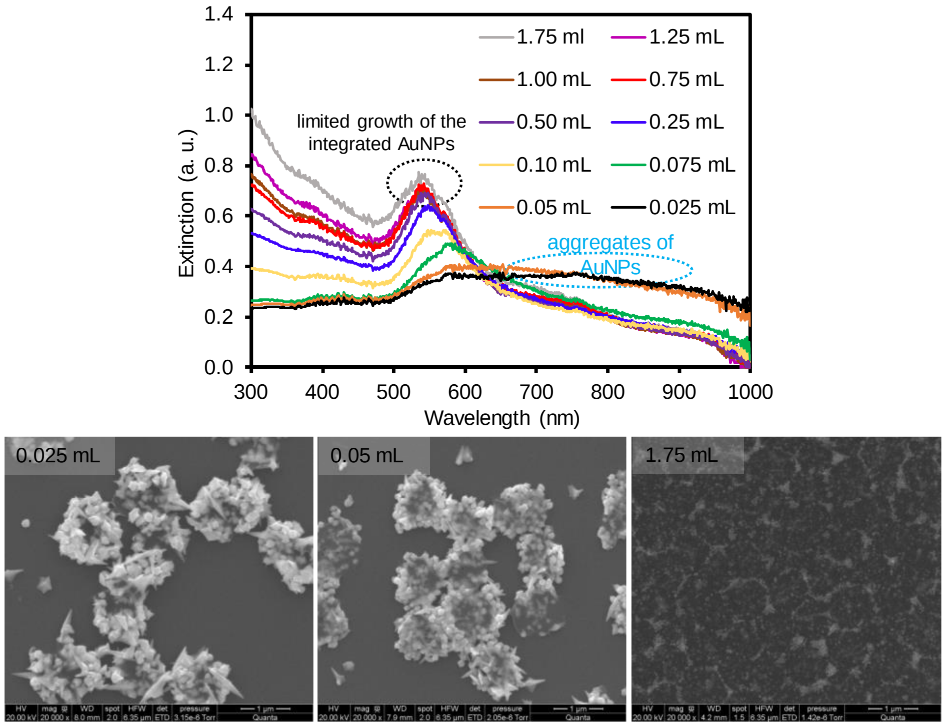

Figure S2. SPR patterns of composite particles prepared by adding various amounts of PNIPAM-AuNP seeds and a few representative SEM images of grown AuNPs across PNIPAM particles prepared above and below the limits.

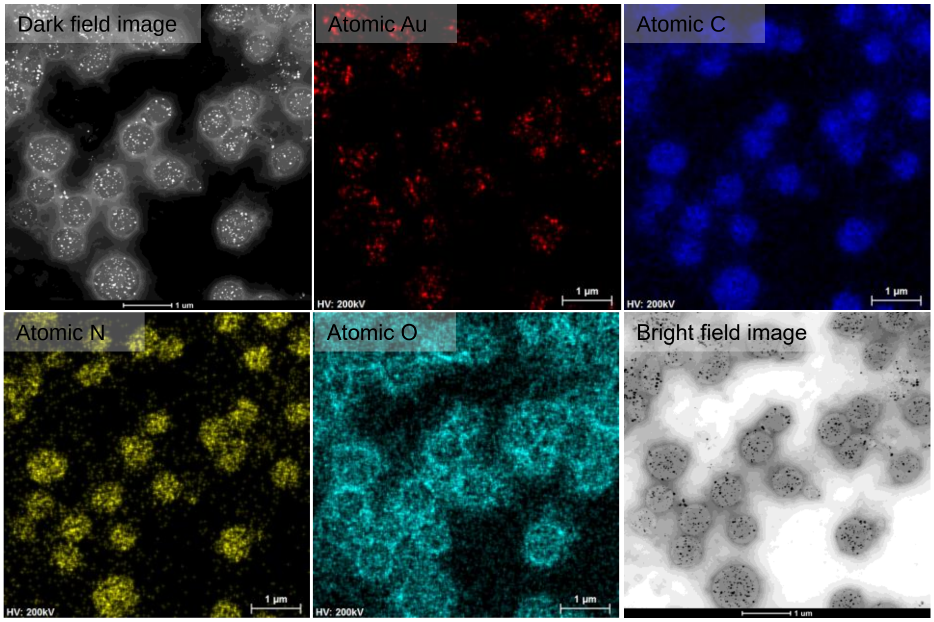

Figure S3. STEM-EDX elemental maps of the representative composite particles prepared with 0.75 mL of the AuNP-PNIPAM seed solution and corresponding dark and bright field images.

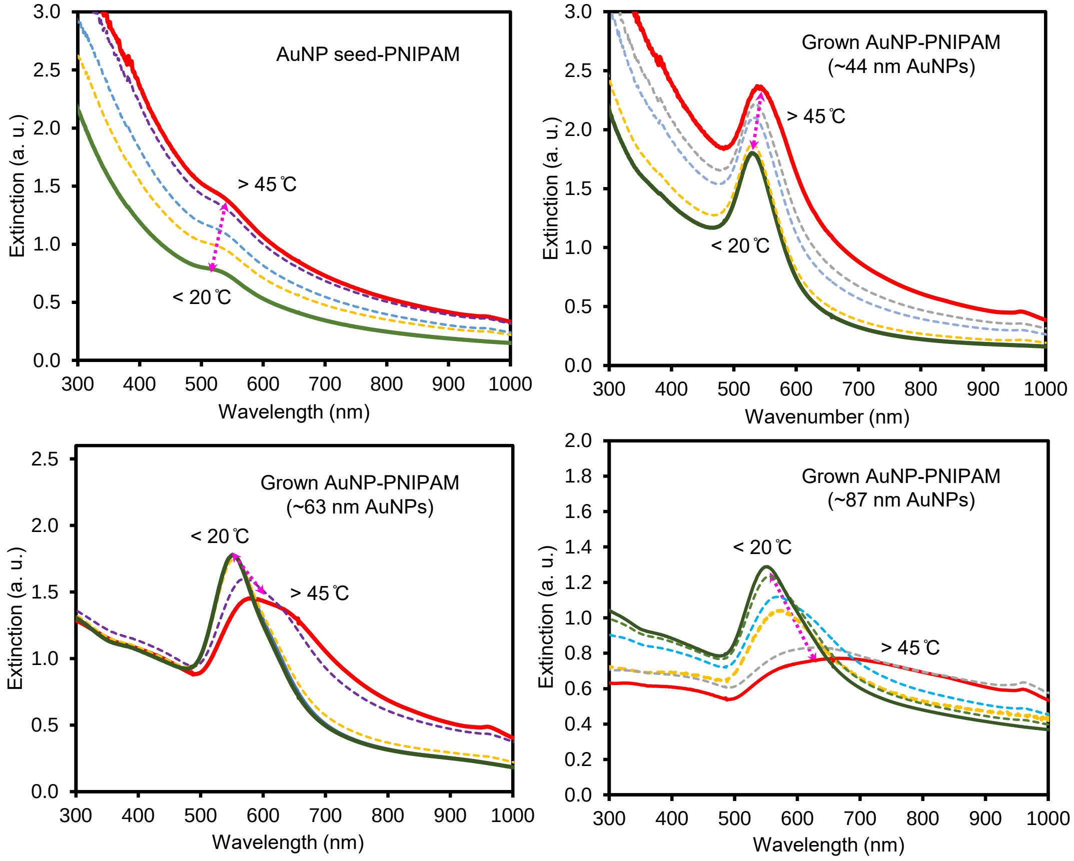

Figure S4. Reversible SPR patterns of various composite particles in water upon heating and cooling the solution temperatures.

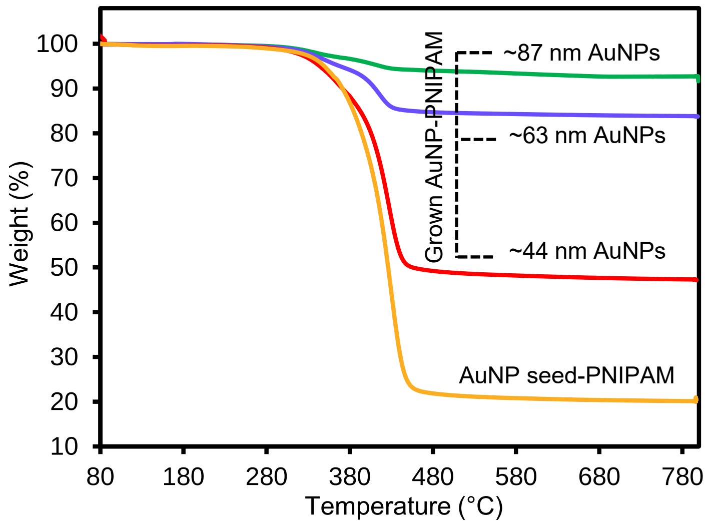

Figure S5. Weight loss patterns of various composite particles using TGA.

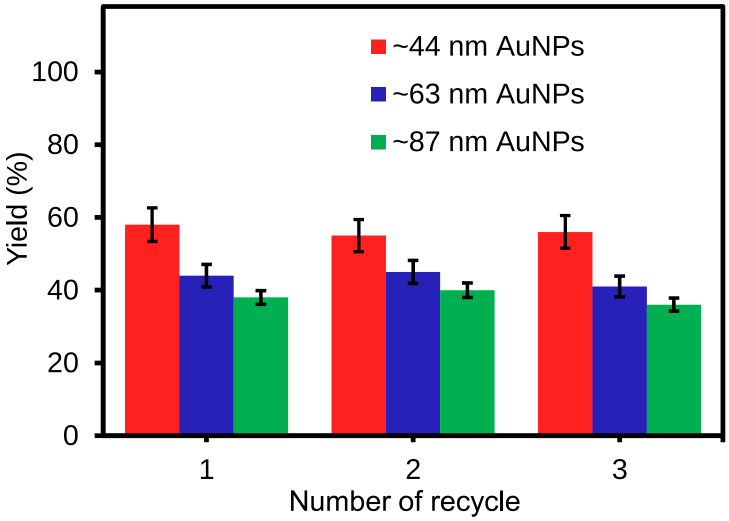

Figure S6. Recyclability of the composite particles in the catalytic homocoupling reaction.

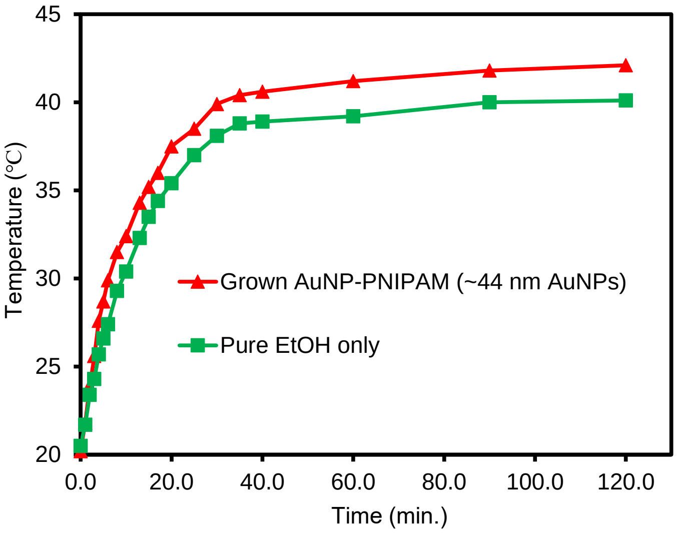

Figure S7. Temperature profiles of pure EtOH and grown AuNP-PNIPAM particles.

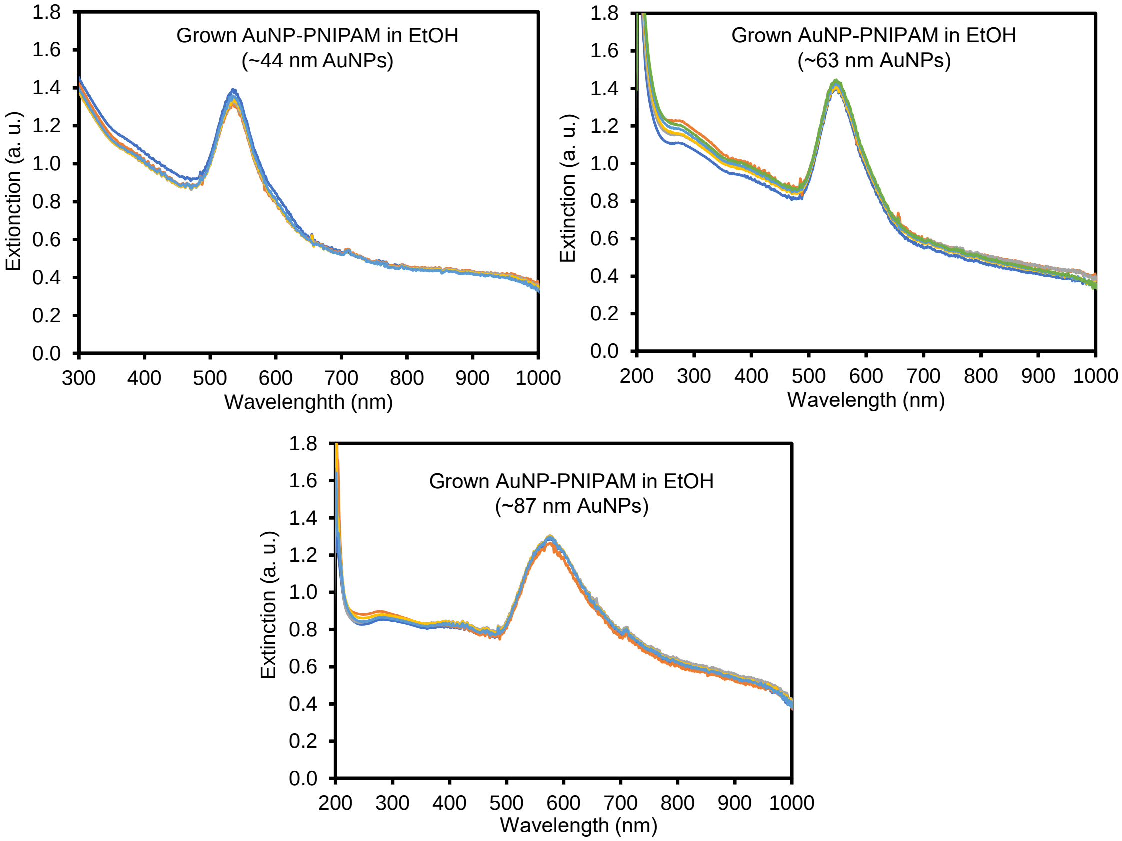

Figure S8. SPR patterns of various composite particles in EtOH upon heating and cooling the solution temperatures (from below 20 ℃ to over 45 ℃).

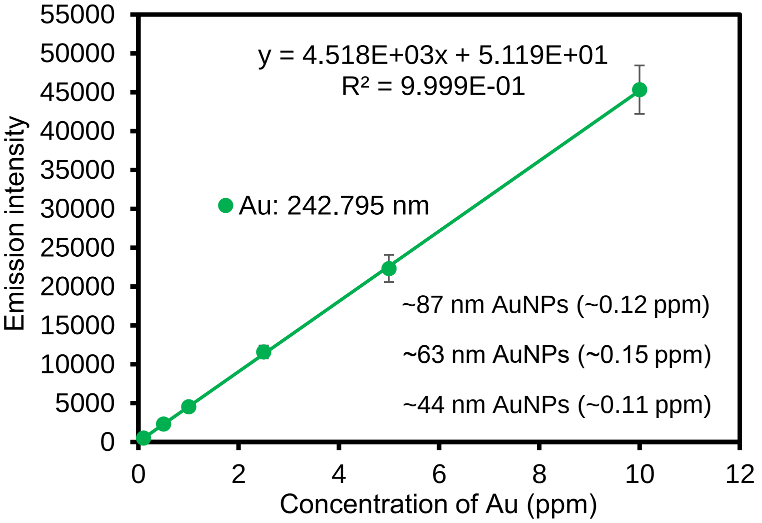

Figure S9. Calibration curve of ICP gold standards and total loss of gold species after the homocoupling reaction.

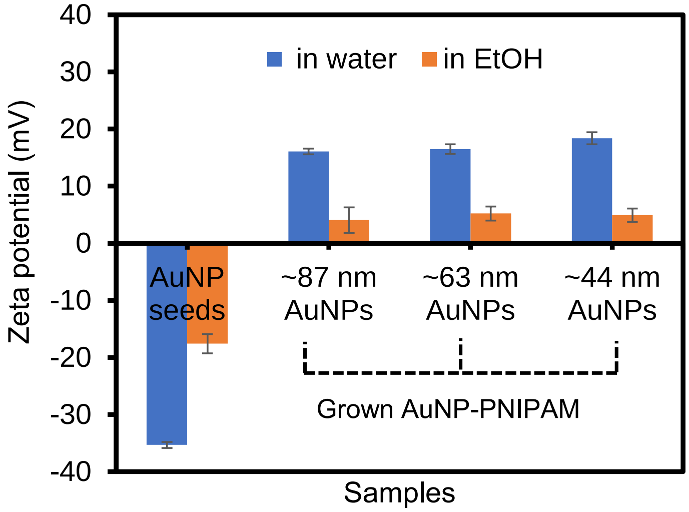

Figure S10. Zeta potentials of various composite particles in water and EtOH.

Supplementary Equation S1. The Debye-Scherrer equation and example calculations for the size of integrated AuNPs.

$d_{hkl} = \frac{k\lambda}{\beta \cos(\theta)}$, where d_{hkl} = mean size, k = 0.89 for shape factor, λ = 0.154 nm for X-ray source, β = full width at half maximum (FWHM) of the peak, and θ = diffraction angle

Composite particles containing AuNP seeds: (111) plane for 2θ = 38.80 (FWHM: 1.06)

$$d_{111} = \frac{k\lambda}{\beta \cos(\theta)} = \frac{0.89 \times 0.154}{radians\,(1.06) \times \cos(radians\,(38.8/2))} = 7.85\ nm$$

Composite particles containing ~44 nm AuNPs: (111) plane for 2θ = 38.76 (FWHM: 0.30)

$$d_{111} = \frac{k\lambda}{\beta \cos(\theta)} = \frac{0.89 \times 0.154}{radians\,(0.30) \times \cos(radians\,(38.76/2))} = 27.75\ nm$$

Composite particles containing ~63 nm AuNPs: (111) plane for 2θ = 38.78 (FWHM: 0.21)

$$d_{111} = \frac{k\lambda}{\beta \cos(\theta)} = \frac{0.89 \times 0.154}{radians\,(0.21) \times \cos(radians\,(38.78/2))} = 39.64\ nm$$

Composite particles containing ~87 nm AuNPs: (111) plane for 2θ = 38.78 (FWHM: 0.14)

$$d_{111} = \frac{k\lambda}{\beta \cos(\theta)} = \frac{0.89 \times 0.154}{radians\,(0.14) \times \cos(radians\,(38.78/2))} = 59.47\ nm$$
